# Supplementary material for: The genome sequence of the Leafhopper, Cicadella viridis (Linnaeus, 1758) (Hemiptera: Cicadellidae)
Source: Wellcome Open Res. 2025 Sep 10;10:502. [Version 1] doi: 10.12688/wellcomeopenres.24866.1 (PMC12485336; doi:10.12688/wellcomeopenres.24866.1)
Supplement: Peer review history

# Reviewer report: Reviewer response for version 1

**DOI:** 10.21956/wellcomeopenres.27390.r132905

**Published:** 2025-09-30

**Authors:** Riccardo Piccinno

**Affiliations:** 1 University of Pavia, Pavia, Italy

The data note "The genome sequence of the Leafhopper, *Cicadella viridis* (Linnaeus, 1758) (Hemiptera: Cicadellidae)" provides a clear description of the genome assembly of *Cicadella viridis*, a widespread leafhopper species in Europe. A female specimen was used for the assembly, which resulted in a chromosome-level genome of approximately 1.4 Gb, obtained by combining long-read sequencing with Hi-C short-read sequencing. The mitochondrial genome was also assembled. BUSCO analysis confirmed the high quality of the assembly.

 Overall, the work is excellent and well presented. The authors employed an up-to-date, standardized workflow of techniques and tools to achieve a high-quality genome assembly, which is particularly impressive given the relatively small size of the insect under study.

 I suggest only a few minor changes:

 Methods section: (1) Do you know the sex of the second speciment used for Hi-C? (2) In the peer review form, I marked the methodological details as only “partly sufficient” because I believe there is room to add a bit more information. For example, the steps necessary for achieving chromosome-level resolution (as reported in the results: “Most of the assembly sequence (99.3%) was assigned to 11 chromosomal-level scaffolds, representing 10 autosomes and the X sex chromosome. These chromosome-level scaffolds, confirmed by Hi-C data, are named according to size”) could be explained more explicitly in the methods. (3) The ASCC pipeline used for decontamination would benefit from a brief description. 

 Introduction: While this is a data note, including a short overview of the species’ biology, particularly its life cycle and ecological impact, would strengthen the paper and be of interest to readers. As a curiosity, the reported distribution outside Europe seems unusual. Is the species’ presence in Madagascar and the eastern Palearctic region known to be recent, possibly linked to globalization, or does it reflect relict populations that survived a past extinction event? A brief clarification would be appreciated in the introduction IF this is known.

Are sufficient details of methods and materials provided to allow replication by others?

Partly

Is the rationale for creating the dataset(s) clearly described?

Yes

Are the datasets clearly presented in a useable and accessible format?

Yes

Are the protocols appropriate and is the work technically sound?

Yes

Reviewer Expertise:

Molecular ecology and evolution of animals, in particular population genomics, metagenomics, genomics, transcriptomics

I confirm that I have read this submission and believe that I have an appropriate level of expertise to confirm that it is of an acceptable scientific standard.

# Reviewer report: Reviewer response for version 1

**DOI:** 10.21956/wellcomeopenres.27390.r132907

**Published:** 2025-09-30

**Authors:** Abdoallah Sharaf

**Affiliations:** 1 University of Konstanz, Konstanz, Germany

The article is scientifically sound in its current form, with no major flaws in rationale, methods, reproducibility, or data presentation. All evaluation questions are answered "Yes," indicating high quality and reliability. No points must be addressed to achieve scientific soundness, as the work already meets rigorous standards for genomic publications. Minor enhancements suggested below could further polish it, but they are not essential. This dataset will be a valuable resource for hemipteran research and biodiversity genomics.

**Recommendations**

 The article is a strong contribution to insect genomics, with excellent integration of ecological context, methodological detail, and data transparency. Strengths include the use of advanced sequencing for a challenging genome (large size, high repeats) and alignment with major biodiversity initiatives.

 While the article is already scientifically sound, the following constructive suggestions could enhance it further:

- Expand the rationale by briefly discussing potential applications, such as using the genome for studying insecticide resistance in leafhoppers or comparative analyses with pest species in Cicadellidae.
- In methods, incorporate a supplementary table summarizing key parameters (e.g., PCR conditions, exact lysis buffers) to complement protocols.io links, reducing dependency on external resources.

Are sufficient details of methods and materials provided to allow replication by others?

Yes

Is the rationale for creating the dataset(s) clearly described?

Yes

Are the datasets clearly presented in a useable and accessible format?

Yes

Are the protocols appropriate and is the work technically sound?

Yes

Reviewer Expertise:

genomics, bioinformatics, and biodiversity research

I confirm that I have read this submission and believe that I have an appropriate level of expertise to confirm that it is of an acceptable scientific standard.
